# In Situ Investigation of the Mechanical Property Anisotropy of TC11 Forgings Through Electron Backscatter Diffraction

**DOI:** 10.3390/ma18102384

**Published:** 2025-05-20

**Authors:** Qineng Li, Ke Li, Wuhua Yuan

**Affiliations:** 1 College of Materials Science and Engineering, Hunan University, Changsha 410082, China; 17886978856@163.com; 2 China National Erzhong Group Deyang Wanhang Die Forging Co., Ltd., Deyang 618013, China

**Keywords:** anisotropy mechanism, in situ tensile, TC11 titanium alloy, CRSS ratio, texture

## Abstract

Electron backscatter diffraction and scanning electron microscopy were performed herein to in situ investigate the influence of texture on the anisotropic deformation mechanism of TC11 forged components. The in situ tensile specimen was cut from the TC11 ring forging, and the tensile force–displacement curve was recorded while the slip lines in the specimen surface detected was traced during the in situ tensile test. The tensile results show that the yield and ultimate tensile strengths decreased in the order of transverse-direction (TD) > rolling-direction (RD) > normal-direction (ND) samples. The anisotropy of the tensile strength was related to the differences in the activated slip systems of the ND, TD, and RD samples. The slip lines results show that in the yielding stage, the ND, TD, and RD samples were dominated by Prismatic <a>, Pyramidal <c + a>, and Pyramidal <a> slips, respectively. In order to further analyze the relationship between the slip system and the yield strength, an anisotropy coefficient was determined to evaluate the differences in resistances for different activated slip systems, providing a good explanation of the variations in the tensile strength anisotropy. The ratios of the critical resolved shear stress (CRSS) of the basal, Prismatic <a>, primary Pyramidal <c + a>, and secondary Pyramidal <c + a> slip systems in the α phase were estimated to be 0.93:1:1.18:1.05 based on the type, number, orientation of slip activations, and Schmid factor. Moreover, the Prismatic <a> slips primarily occurred in the axial and radial (ND and RD) samples with [0001] and [$\overset{-}{1}2\overset{-}{1}2$] textures, whereas the Pyramidal <c + a> slip system was dominant in the TD samples with [11$\overset{-}{2}2$] and [10$\overset{-}{1}$2] textures. Overall, this research demonstrates that the activation of the α-phase slip depends on the grain orientation, SF, and the CRSS, promoting strong strength anisotropy.

## 1. Introduction

The TC11 titanium alloy (Ti–6.5Al–3.5Mo–1.5Zr–0.3Si), an (α + β)-type titanium alloy with excellent comprehensive performance, was developed in China in the early 1980s based on the BT9 titanium alloy. Among the martensitic α + β heat-resistant titanium alloys, TC11 exhibits the best heat resistance at temperatures below 500 °C. Furthermore, it offers high specific strength, good performance at medium temperatures, and good corrosion resistance. Accordingly, it is widely employed in the aerospace field for manufacturing various components, including compressor discs, blades, fasteners, and ring-shaped parts [1,2,3,4,5]. Notably, forging improves the segregation of titanium alloy ingots while promoting the compaction and bonding of internal pores and loosely organized titanium ingots. These changes increase the metal density and consequently enhance its mechanical properties. As the shape of the titanium ingot changes, the metal experiences relative flow within, forming a regular and directional fiber arrangement within the metal. This arrangement makes the mechanical properties of the metal directional. Knowledge of the radial and circumferential properties of ring-shaped titanium parts is important for various industrial applications. Therefore, to ensure the quality of forged parts, investigation of methods for anisotropy elimination is necessary [6,7,8,9,10]. The prerequisite for eliminating the anisotropy of the mechanical properties of the forged parts is to understand their generation mechanism. Deformation is important in thermal–mechanical processing and can be exploited to change and form specific textures [11,12]. Research has shown that forging exerts a notable influence on the micro texture of the IMI8-34 alloy [13]. In order to unravel the relation between the organization and performance of titanium alloys, some analytical techniques and research methods have been developed. L. Germain et al. proposed a scientific method that combines electron backscatter diffraction (EBSD) data with BSE images to separate the micro texture of the secondary α from the transformed beta phase [14,15]. The texture evolution of the Ti–6Al–4V plate with dual-phase organization was studied by A. Slam et al. through EBSD/energy-dispersive X-ray spectroscopy measurements. The textures of the two types of α grains were successfully separated in their work, then a conclusion was formed that the texture of the α grains is aligned with the rolling direction (RD) [16].

Moreover, the occurrence of slips within favorably oriented grains (called ‘soft grains’) causes the deflection of the base plane from the applied tensile axis during the deformation, accompanied by strain accumulation around the neighboring grains’ boundary. Therefore, under tension, short cracks eventually form owing to the shear stresses in those ‘hard orientation’ grains. This kind of phenomenon eventually promotes the formation of slip bands, which gradually turn into short cracks under uniaxial loading [17]. Research has gradually revealed that the strength anisotropy is quite closely related to the Schmid factor (SF), as well as the critical resolved shear stress (CRSS), of the activated system. In particular, the soft and hard orientations, as well as the slip anisotropy, explain the uneven strain on the grain boundaries, which then leads to local triaxial stresses [18]. Some researchers believed that the mechanical property anisotropy of titanium alloys is dependent on the Schmid factor of the activated Prismatic slip system, which combines the microscopic mechanism and mechanical properties well. Lei’s group [19] evaluated the SF of the activated Prismatic system to predict the yield anisotropy ratio. Nevertheless, there are also reports about the basal slips and how they play a role during the deformation process of dual-phase titanium alloy [20].

Their method is quite effective for explaining the anisotropy of tensile properties attributed to the slip systems. For example, when studying the anisotropy of tensile properties of α titanium alloy with the strong rolling texture, He et al. [21] found that the plastic deformation along the TD is primarily based on the substrate slip. However, the contribution of the pyramidal slip activation to the yield strength was completely ignored. It is newly reported that the contribution of the activated Pyramidal <c + a> slips to the tensile properties cannot be ignored, as a relatively high CRSS is required to activate the Prismatic systems, even though the proportion of which is small [22,23]. Therefore, an anisotropic evaluation method that considers all activated slip systems is needed. Jia R. [23] investigated the mechanical anisotropy of Ti60 plates along the RD, TD, and 45° direction and found that quite a few pyramidal <c + a> slip systems are successfully activated along the TD during in situ tensile engineering. This is due to the high CRSS required to activate such slip systems, as well as the low SF. This explains why the highest yield strength was observed in the TD samples.

The above research indicates that texture has a notable impact on deformation and crack nucleation mechanisms. Therefore, conducting in-depth research on texture to further understand its role in deformation and avoid sudden failure during service is imperative [17,18,19,20,21,22,23,24,25]. However, the tensile samples of the in situ tensile experiments conducted by the aforementioned research institute were all located on the same plane, namely, the xoy plane in three-dimensional space. Due to the different metal flow conditions of the ring forgings along different directions during the die forging process, the grain shape and orientation along different directions are eventually formed, which ultimately has an impact on the mechanical properties of the forgings along ND, RD, and TD. In our study, the three directions of performance testing for forgings correspond to the ND, RD, and TD.

Based on the research of Long and Zhang et al. [22,26], compared with near-β titanium alloys and α titanium alloys, the dominant phase in the deformation process is determined by the content of each phase. Long et al. [26] conducted an in situ study of TC21 dual-phase titanium alloy and found that the α phase had a greater influence during its deformation. Based on the fact that TC11 titanium alloy has a large proportion of α phases at room temperature, this study mainly studies the slip system and texture of α phases. This paper considers TC11 ring forgings annealed under the thermal treatment of 960 °C-1 h-AC + 530 °C-6 h-AC as the research object, and samples are obtained along the RD, ND, and TD. The activated slip systems of each sample in the yield and hardening stages are analyzed through in situ tensile tests. At the same time, the CRSS ratio and the deformation resistance coefficients are proposed to further explain the yield strength anisotropy. Further, EBSD analysis is conducted on three samples with different stretching positions to study the α texture characteristics after forging-induced deformation and the selection tendency of each slip system. The correlation among the deformation texture, slip system activation, and yield strength was comprehensively analyzed.

## 2. Materials and Methods

### 2.1. Material

Dual-phase TC11 alloy forgings were provided by China National Erzhong Wanhang Die Forging Co. (Deyang, Sichuan province, China), which composition is Ti-6.5Al-3.5Mo-1.5Zr-0.3Si. They comprised the α phase (hexagonal close-packed structure, hcp) with a volume fraction of 92% and the β phase (body-centered cubic structure, bcc) with a volume fraction of 8%. Further, they have a biphasic structure where the equiaxed primary α phase (thickness: 10 μm) constituted 40% of the volume fraction, and the secondary α_s_ plate (thickness: 100 nm) embedded in the β matrix in a layered structure constituted the remaining 60%.

### 2.2. Specific Surface Preparation and Conditions for the EBSD Measurements of the Micro Tensile Samples

**Figure 1:**
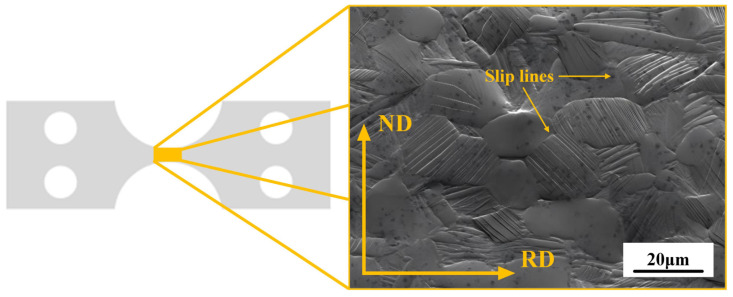
Slip lines generated during in situ tensile tests of RD specimen.

Samples were processed by electrical discharge corrosion, followed by mechanical polishing. In particular, to obtain sufficient EBSD patterns on such fine titanium alloys, any stress or residual deformation in the surface layer resulting from the mechanical polishing must be removed. In the next step, the electrolytic polishing is performed. The final step enabled the scanning electron microscopy observation of the slip bands with sufficient surface quality. Figure 1 illustrates the positioning of slip lines on the surface of the in situ stretched samples.

EBSD analyses were performed using an Oxford Nordly max3 field emission scanning electron microscope (Oxford instruments, Oxford, UK) operated at a voltage of 20 KV with a scanning step of 2 µm. Notably, researchers focused mainly on α phase, as the phase is considered to primarily support plastic deformation. EBSD data were collected using the Channel5 software (Oxford instruments HKL A/S 2007, Ver.5.0.9.0) (Oxford instruments, Oxford, UK). The texture of the scanning area was characterized by the pole figure.

### 2.3. In Situ Tensile Test

**Figure 2:**
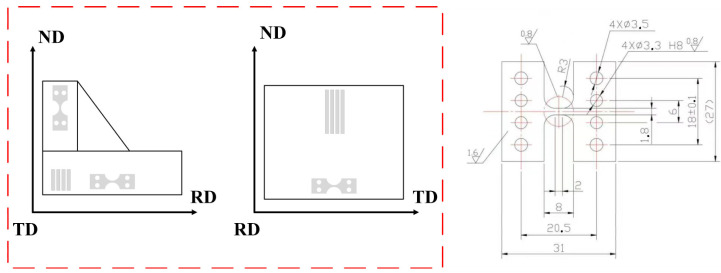
Slip lines generated during in situ tensile tests.

Under mechanical load, the deformation at the microscale was characterized by using a machine that can not only operate in situ tensile tests but also has a scanning electron microscope. Several flat plates (size 31 mm × 27 mm × 2 mm) were cut along three directions of the forging and polished to prepare the specimens for the tensile tests (Figure 2). This machine allows for uniaxial tensile testing, with a maximum displacement of 10 mm for the jaws and a variable rate between 0.03 and 1.4 mm/min. The results presented in this article were obtained at displacement rates of 0.05 mm/min, which allow for easy termination of the experiment at predetermined stress or strain levels. The test can be paused by stopping the motor and capturing images of the microstructure at any time. This paper will determine the strain intervals corresponding to the yield stage and work hardening stage based on the engineering stress–strain curve of TC11 titanium alloy, and set the imaging points for in situ tensile SEM and EBSD observations accordingly based on these findings. Previous in situ tensile tests of dual-phase titanium alloy have shown that the envelope of the tensile curve obtained for experiments with interruptions fits correctly with the curve obtained for uninterrupted experiments [26].

### 2.4. Determination of the Activated Slip System

Recently, a useful method for determining activated slip systems based on slip trajectories was proposed by some scholars [24,27]. Two criteria for determining the activation of slips were established. The first is that in the equivalent slip system, the slip system that has the largest SF is the most easily activated. The second is that, once the slip line on the surface of the specimen’s actual trace angle is observed, the theoretical trace angle calculated for the activated slip system has to be the closest. Accordingly, all slip systems that are activated were determined as follows.

The first step involves calculating the SF coefficients to choose the slip system that has the highest SF coefficient from all four slip system groups. The second step involves calculating a theoretical rotation angle (θ_th_) between the intersection line on the sample surface and the selected slip system’s slip plane, as well as the tensile direction. The final step involves measuring the true rotation angle (θ_re_) between the slip line on the specimen’s surface and the tensile direction. In the meantime, the error between θ_th_ and θ_re_ is calculated, and the activated slip system equals the slip system with the lowest error. Compare the measured θ_re_ with the calculated θ_th_ values to identify the most appropriate slip system. The identification follows two criteria: (1) The absolute value of θth for the activated slip system should closely match the measured θre. (2) Among all candidate slip systems, the activated slip system must exhibit the highest Schmid factor. The SF and θ_th_ can be obtained through the following steps of calculation, known as the unit vector of each slip system. The (hkl) [uvw] is calculated using the following equation:

(1) $$\vec{n}=\frac{\left(\frac{h}{a},\frac{k}{b},\frac{l}{c}\right)}{\sqrt{{\left(\frac{h}{a}\right)}^{2}+{\left(\frac{k}{b}\right)}^{2}+{\left(\frac{l}{c}\right)}^{2}}}$$

(2) $$\vec{s}=\frac{\left[ua,vb,wc\right]}{\sqrt{{\left(ua\right)}^{2}+{\left(vb\right)}^{2}+{\left(wc\right)}^{2}}}$$

where $\vec{s}$ and $\vec{n}$ are the unit normal vectors of the slip direction and the slip plane, respectively. The a, b, and c (lattice parameters) values of the α phase are 0.295, 0.295, and 0.468 nm, respectively. The stretching direction (T^s^) in the sample coordinate system is converted into the corresponding vector direction (T^c^) in the crystal coordinate system according to the following equation:

(3) $$T^{C}=gT^{S}$$

(4) $$g=\left(\begin{array}{ccc} cos\varphi_{1}cos\varphi_{2}-sin\varphi_{1}sin\varphi_{2}cos\Phi & sin\varphi_{1}cos\varphi_{2}+cos\varphi_{1}sin\varphi_{2}cos\Phi & sin\varphi_{2}sin\Phi \\ -cos\varphi_{1}sin\varphi_{2}-sin\varphi_{1}sin\varphi_{2}cos\Phi & -sin\varphi_{1}cos\varphi_{2}+cos\varphi_{1}sin\varphi_{2}cos\Phi & cos\varphi_{2}sin\Phi \\ sin\varphi_{1}sin\Phi & -cos\varphi_{1}sin\Phi & cos\Phi \end{array}\right)$$

where (φ1, Φ, and φ2) are the Euler angles collected from the EBSD data, and the Ts is [100] in this research. Thus, each slip system’s SF can be represented by Equation (5); θ_th_ can be calculated by Equation (6):

(5) $$SF=\left(n\cdot T^{C}\right)\left(s\cdot T^{C}\right)$$

(6) $$\theta_{th}=arccos\left\{\left(ge^{s}\times n\right)\cdot T^{C}\right\}$$

Here, in the sample coordinate system, es is the unit normal vector of the sample surface, i.e., the (001) plane. The symbols ‘⋅’ and ‘×’ denote the dot and vector products, respectively.

## 3. Results

### 3.1. Microstructure and Mechanical Properties of the Specimen Before In Situ Tensile Test

**Figure 3:**
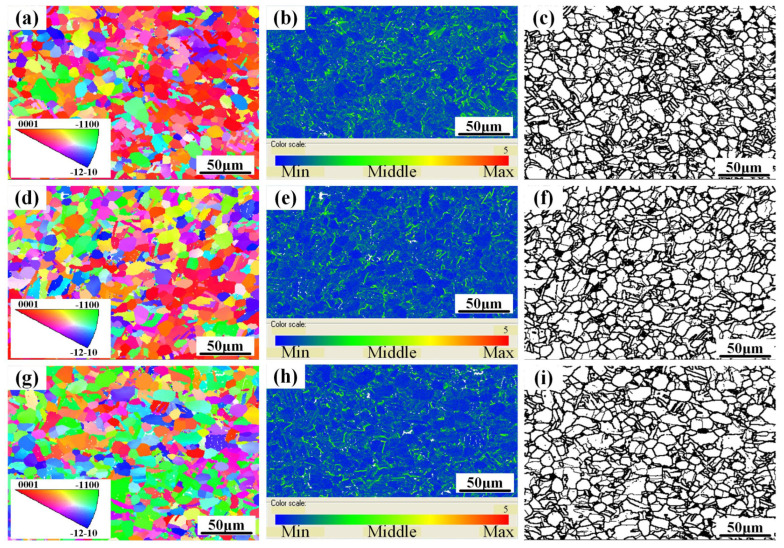
Inverse pole figure, local misorientation, and grain boundary plot along the three directions before in situ tensile test: (**a**–**c**) ND; (**d**–**f**) RD; (**g**–**i**) TD.

Figure 3 shows the inverse pole figure, local misorientation, and grain boundary plot along the ND, RD, and TD before the in situ tensile test. After heat treatment of TC11 titanium alloy forgings, the overall grain orientation of specimens in different directions exhibits significant variations. Specimens along the transverse direction (TD) demonstrate a relatively higher proportion of grains with the {−1100} orientation (Figure 3a,d,g). Additionally, the misorientation angles between grains in different directions show minimal differences, indicating limited variations in dislocation density across the three orthogonal directions (Figure 3b,e,h). Furthermore, the grain size remains relatively uniform along different directions (Figure 3c,f,i).

**Figure 4:**
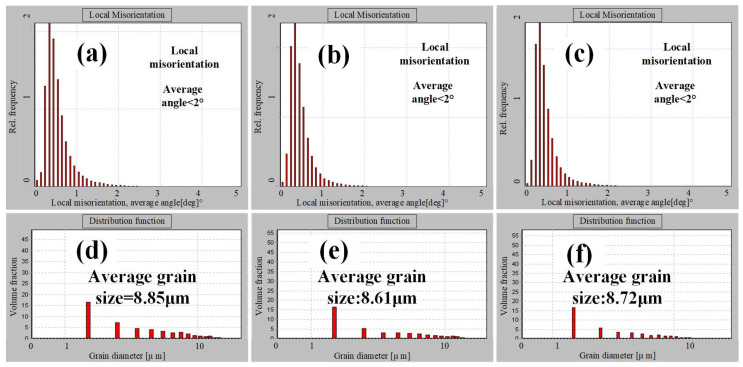
Histogram of local orientation and grain size distribution of TC11 titanium alloy forgings along three directions: (**a**,**d**) ND; (**b**,**e**) RD; (**c**,**f**) TD.

Figure 4 shows histograms of the local misorientation and grain size distribution. From Figure 4a–c, it can be observed that the grain orientation differences in TC11 titanium alloy forgings along the ND, RD, and TD directions are extremely small, with rare occurrences of abrupt large misorientation grains. These findings indicate that the dislocation densities are similar, theoretically suggesting that specimens along all three directions would require comparable accumulated deformation levels for crack initiation caused by dislocation pile-up during tensile deformation. In Figure 4d–f, the overall grain size distribution of the forgings appears relatively uniform, while the finer grains along individual directions result in a lower likelihood of grain refinement strengthening.

**Figure 5:**
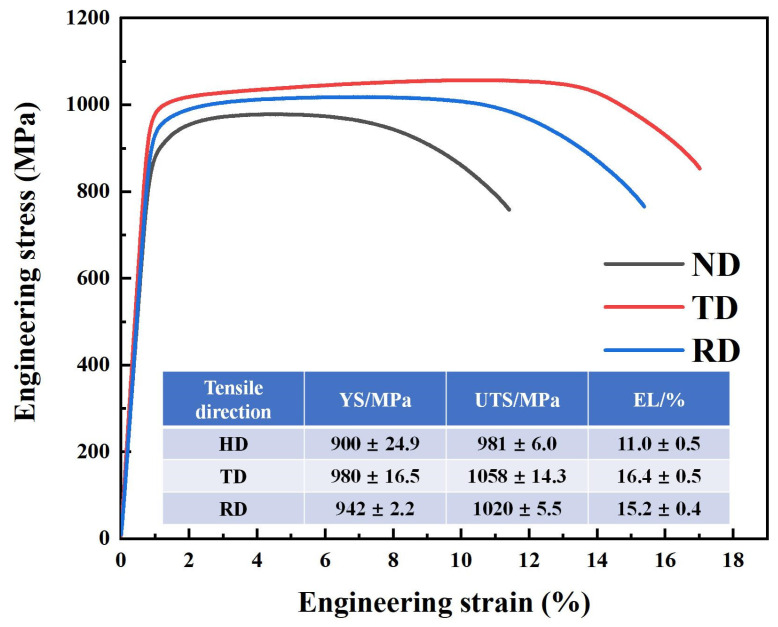
The room-temperature mechanical properties of TC11 titanium alloy forgings along ND, RD, and TD.

**Figure 6:**
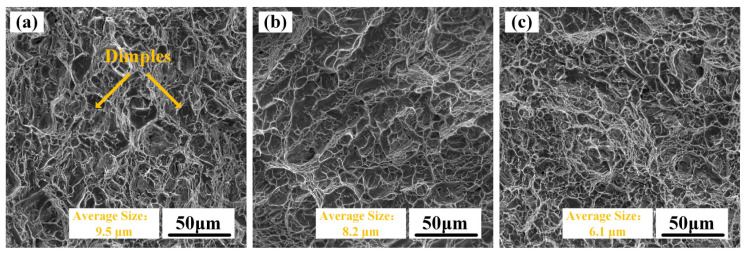
Fracture surfaces of tensile specimens taken along different directions in TC11 titanium alloy forgings: (**a**) ND; (**b**) RD; (**c**) TD.

The room-temperature tensile properties of TC11 titanium alloy forgings along the ND, RD, and TD directions are shown in Figure 5. These tensile data were obtained through a universal mechanical testing machine. The TD specimens exhibited the highest yield strength, tensile strength, and elongation, followed by RD specimens, while ND specimens showed inferior performance, demonstrating significant strength and plasticity anisotropy. Unlike the typical strength–plasticity relationship observed in common titanium alloys, the TD specimens exhibited both the highest strength and optimal plasticity. Fracture analysis of the three tensile specimens yielded consistent conclusions (Figure 6a–c). The fracture modes of ND, RD, and TD specimens all showed typical microvoid coalescence characteristics, with average dimple sizes following the sequence ND > RD > TD. Smaller dimple sizes correspond to better material plasticity, which aligns perfectly with the observed tri-directional plasticity trend in forgings: TD > RD > ND.

### 3.2. In Situ Tensile Test

**Figure 7:**
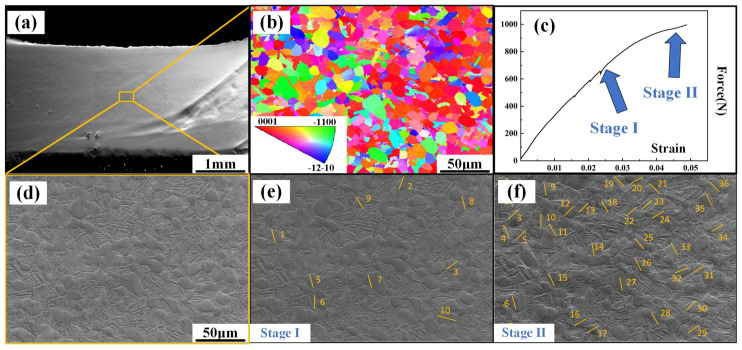
In situ tensile observation of the samples along the ND: (**a**) original image of tensile specimen, (**b**) inverse pole figure of tensile specimen before in situ tensile test, (**c**) Force–strain curve, (**d**) in situ image before tensile test, (**e**) in situ image at stage I, and (**f**) in situ image at stage II.

Figure 7 shows the in situ tensile image of the ND sample. Figure 7a illustrates an original image of the sample surface. The primary grains are compressed during the deformation process and deform along the direction perpendicular to the compression direction. The Euler angle and crystal orientation can be obtained from the EBSD diagram employed to identify activated slip systems (Figure 7b). Figure 7c records the stress–strain curve of the in situ tension, where each fluctuation represents an observation. During the in situ stretching process, two notable stages can be observed: stage I and stage II, referring to the yielding and hardening stages, respectively (Figure 7d,e). In stage I, only a few slip lines can be well observed, while in stage II, it is clearly shown that more grains begin to slip. In Table 1, all the motion slip systems in both of the two stages in Figure 7a have been precisely counted and are listed. Notably, during the yield and hardening stages of the ND samples, the Prismatic slip dominates the tensile process. Meanwhile, although the critical shear stresses for the Prismatic <a> and basal <a> slip systems are lower than those for the Pyramidal <c + a> and Pyramidal <a> slip systems, the basal <a> slip systems are rarely activated, even during stage II (hardening stage). Additionally, in stage II, the proportion of Pyramidal <c + a> and Pyramidal <a> slips substantially increases. In stage I, most of the favorable orientation slip systems activated first gradually rotate towards the ‘hard orientation’ state, after which the Pyramidal <a> slip is activated to adapt to deformation, which might be the main reason for the phenomenon above.

**Figure 8:**
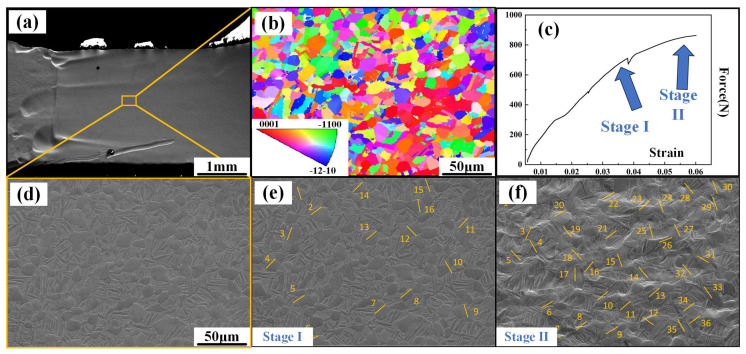
In situ tensile observation of the samples along the RD: (**a**) original image of tensile specimen, (**b**) inverse pole figure of tensile specimen before in situ tensile test, (**c**) Force–strain curve, (**d**) in situ image before tensile test, (**e**) in situ image at stage I, and (**f**) in situ image at stage II.

Figure 8 shows the in situ stretching image along the RD. The original image of the deformation area is vividly shown in Figure 8a, and the EBSD chart is shown in Figure 8b. Stages I and II correspond to the yielding and hardening stages, respectively. The slip marks appear neatly organized at the beginning of the slip in Figure 8d. As the stretching continues, the slip marks in the hardening stage exhibit more randomness (Figure 8e). In Table 2, it is clear that the activation of Pyramidal <a> in stage A is an absolute advantage. Notably, the SF of most of the Pyramidal <a> systems is >0.4. The basal slip system, Prismatic slip system, and Pyramidal <c + a> system are more activated during the hardening stage. This is because after particle rotation, some ‘hard-oriented’ particles can be activated, such as some Pyramidal <c + a> systems and Prismatic systems.

**Figure 9:**
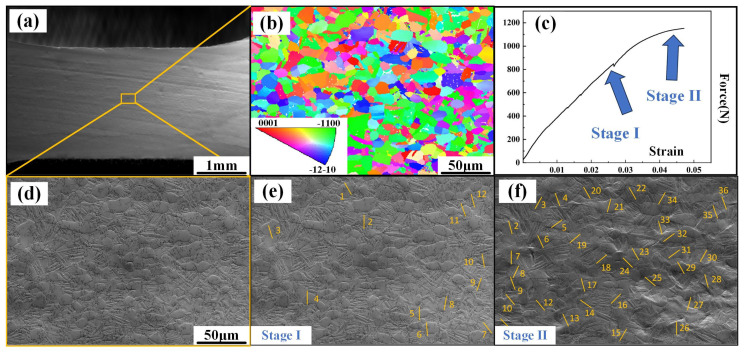
In situ tensile observation of the samples along the TD: (**a**) original image of tensile specimen, (**b**) inverse pole figure of tensile specimen before in situ tensile test, (**c**) Force–strain curve, (**d**) in situ image before tensile test, (**e**) in situ image at stage I, and (**f**) in situ image at stage II.

Figure 9 shows the in situ tensile image of the TD samples. Figure 9a–c show the original image, EBSD image, and in situ tensile curve of the observation area, respectively. The primary α particles elongate along the TD, perpendicularly to the deformation direction. In the hardening stage in Figure 9, the slip marks appear more disordered. From the identified slip system results (Table 3), more Pyramidal <c + a> slips that have high SFs were activated in stage I for the TD samples, compared with the case for the ND samples. A few basal and Prismatic slip systems with lower SFs were found in stage I, and the number of Pyramidal <a> and Pyramidal <c + a> slips activated in stage II was reduced. As Stretching continues, more basal and Prismatic slip systems are activated, most of which are in a ‘hard orientation’ state because of their low SF coefficients. Therefore, the increase in the number of Prismatic and Basal slip systems activated is caused by the neighboring particles activated earlier. During the deformation process, grains with good orientations are activated in stage I. During the tensile process, once the uniaxial deformation reaches a certain degree, dislocations accumulate at adjacent grain boundaries, causing the concentration of stress. To adapt to the deformation, the external slip amplitude of the particle slip systems with the ‘hard orientation’ may be smaller than that in stage I. Generally, the activation ratios of the Pyramidal slip <a> and Pyramidal slip <c + a> are high, indicating that a large external force is required for deformation to occur along the TD.

**Figure 10:**
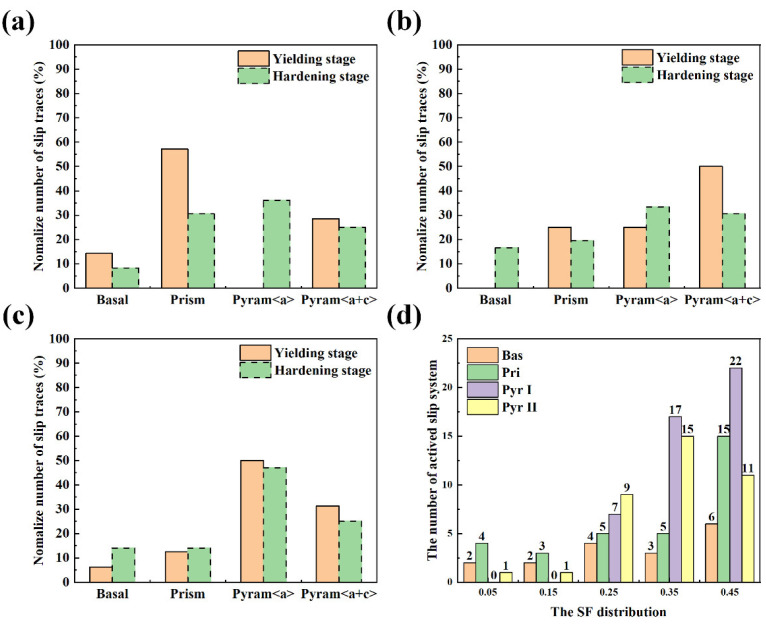
Normalized number of slip traces for the (**a**) ND, (**b**) TD, and (**c**) RD samples; (**d**) the SF distribution of the activated slip systems.

Figure 10 illustrates the differences in the activations of the slip systems in each stage and direction. First, compared with that in the hardening stage, the anisotropy of slip activation in the yield strength stage is more pronounced. Owing to the relatively low rotation of the crystal orientation in the yield strength stage, the original crystal orientation is primarily retained. During the hardening stage, the rotation of grains is driven by considerable deformation, causing some grains to rotate towards other good orientations. In that case, new slip systems can be activated [28]. Second, it can be concluded that the activation of the slip system during the yielding stage considerably varies with changes in the stretching direction (Table 4).

For the ND samples, the basal <a> slips negligibly occur, and the Prismatic <a> slips are easily activated by weak external forces. The Pyramidal <a> and Pyramidal <c + a> slip planes can easily achieve high SF coefficients and can be activated by relatively weak external forces. Therefore, the Prismatic <a> and Pyramidal <a> slips are established as primary modes, whereas the Pyramidal slip <c + a> serves as the secondary mode. Contrarily, for the TD and RD samples, the stretching direction is almost parallel to the c-axis, making it difficult to activate the negative-angle planes of the basal plane <a> and Prismatic <a> slip systems. For the RD samples, the Pyramidal <a> slip is preferentially activated, and for the TD samples, the proportion of Pyramidal <a> and Pyramidal <c + a> slips substantially increases, indicating the presence of strong external forces. Therefore, although the yield strength of the RD samples is high, the TD samples exhibit the highest yield strength.

## 4. Discussion

### 4.1. Estimation and Analysis of the CRSS Ratio for the α Phase

The slip behavior of the α phase determines the yield strength of the TC11 forging. In Equation (7), for a single crystal, the yield strength depends on its *SF* and *CRSS* [29]. Generally, equivalent slip systems have a common *CRSS*, whereas non-equivalent slip systems have different *CRSS* conditions. It is quite necessary to evaluate the *CRSS* conditions of the four slip systems in the α phase to provide in-depth information on the strength anisotropy.

(7) $$\sigma_{y}=\frac{CRSS}{SF}$$

Generally, slip activation can be promoted under a high slip ratio. According to all four types of activated slip systems’ *SF* distribution in Figure 7d, with an increasing *SF* coefficient, the activated slip systems’ number clearly has the trend to increase, with approximately 75% of activated slip systems having *SF* values of >0.3. However, even in cases where the *SF* is <0.3, basal and Prismatic <a> slips have an absolute advantage, indicating that the *CRSS* conditions of the α phase are different.

Although fewer basal <a> slips are activated compared with the first order and second-order Pyramidal slips, two facts should be considered: the first is the activation of basal slips with *SF* coefficients of <0.3, and the other is the considerably smaller equivalent slip system family of the base <a> slips compared with that of the Pyramidal <c + a> slips. In contrast, basal <a> slips are more easily activated, indicating that basal slips have a lower CRSS than Pyramidal slips. The *SF* coefficients of the two types of Pyramidal slips are similar, both being >0.3. However, the total number of activations for the Pyramidal <a> slip is slightly higher than that for the Pyramidal <c + a> slip, indicating that the former is more easily activated. Notably, the substrate and Prismatic slips can also be easily activated because of their relatively low SF coefficients, indicating that their *CRSS*s are relatively low.

Researchers [30,31,32,33,34,35] have shown that, regardless of the phase, a common trend is observed for the *CRSS*: increasing from Prismatic < a> to basal <a> to Pyramidal <c + a> slip. The *CRSS* of the conical slip of the α phase is substantially higher than those of the Prismatic and basal slips. As shown in Equation (7), the CRSS could be proportional to the *SF* coefficient while the yield strength remains constant. Therefore, based on the average *SF* coefficient of the different types of activated slip systems in Figure 4, the *CRSS* ratio of the α phase in TC11 forgings can be estimated. The calculation results show that the *CRSS* ratios for the basic <a>, Prismatic <a>, Pyramidal <a> slip, and Pyramidal <c + a> slips are 0.93:1:1.18:1.15, respectively. According to Irvin Séchepée et al. [36], the *CRSS* ratio of TC11 titanium alloys is close to the usual ratio range of dual-phase titanium alloys. Many CRSS ratios are reported in Table 5 with results from other studies. The values of *CRSS* ratios differ from one study to another. Such variations can be attributed to the microstructure studied, its morphology, grain sizes, phase proportions, or even its texture.

### 4.2. Determination of the Resistance Coefficient

The results of the above analyses reveal that there is a strong relation between the slip system and yield strength. Efforts have been devoted to establishing an effective method for predicting yield strength. The activity of base <a>, Prismatic <a>, and Pyramidal slips and the *CRSS* ratios along different directions were quantitatively analyzed and calculated by Bridier et al. [24]. Slips directly lead to differences in the *CRSS* distribution along different directions, which is the fundamental reason for the anisotropy of the tensile properties. To provide an in-depth explanation of the intrinsic anisotropy mechanism, it is necessary to consider the *SF* and the ratio for slip activation of each slip system along different directions. The SF and external force (*σ*) determine the decomposed shear stress (*τ*) of each slip system. The *τ* is calculated using the below equation [47,48].

(8) τ=σμ

The contributions of every slip system to the deformation process were determined using an influence coefficient method [23]. The *SF* resistance coefficient values (*R_μ_* values) of certain slip systems activated in tensile testing, including basal <a>, Prismatic <a>, Pyramidal <a>, and Pyramidal <c + a> slips, can be expressed as follows:

(9) $$R_{\mu }=1-\frac{\mu_{ave}}{0.5}=1-\frac{\sum_{i}^{n}\mu_{i}}{0.5n}R_{\mu }\varepsilon \left(0,1\right)$$

For all slip systems, it is necessary to obtain the weight coefficient values (*R_f_* values) of each slip system during the in situ tensile test to consider each one’s contribution. The *R_f_* values of each slip system are determined using the following equation:

(10) $$R_{f}=\frac{n^{slip}}{n^{all}}{\;\;\;R}_{f}\varepsilon \left(0,1\right)$$

Therefore, the chi value can be adopted to describe the difficulty of activating any slip system. For α + β titanium alloys, the ratio of the resolved shear stress (τ) of each slip system has been reported as τ_Basal_: τ_Prismati_c: τ_Pyramidal_-1st: τ_Pyramidal_-2nd = 1:1:3:3 [18,49]. When given the *CRSS* (τ), the *R_μ_* and *R_f_* of the *SF* of each slip system can be expressed as:

(11) $$\chi =\tau \cdot R_{\mu }\cdot R_{f}$$

Evidently, basal and Prismatic slips can be activated by very weak forces (τ). However, a larger decomposition shear stress (τ) is necessary for activating Pyramidal <a> and Pyramidal <c + a> slips. Therefore, this parameter χ represents the resistance that activates all slip systems along any certain stretching direction, which is calculated from the following formula:

(12) $$\chi =\tau_{CRSS}^{basal}\cdot R_{\mu }^{basal}\cdot R_{f}^{basal}+\tau_{CRSS}^{prism}\cdot R_{\mu }^{prism}\cdot R_{f}^{prism}+\tau_{CRSS}^{pyram-1st}\cdot R_{\mu }^{pyram-1st}\cdot R_{f}^{pyram-1st}+\tau_{CRSS}^{pyram-2st}\cdot R_{\mu }^{pyram-2st}\cdot R_{f}^{pyram-2st}$$

Combining Equations (8)–(12), Table 6 presents the calculation results of the χ values for the RD, ND, and TD samples, corresponding to 0.73, 0.58, and 0.76, respectively. Overall, a downward trend is observed, i.e., TD > RD > ND, which is consistent with the strength performance trend. Noticeably, the ND samples have the lowest chi-square value, indicating that a large proportion of the slip system can be activated by weak external forces. This implication is supported by the relatively low yield (922 MPa) and ultimate tensile strengths (1028 MPa) observed for the ND specimens. Contrarily, the chi value for the TD samples is the highest among the three directional samples, indicating the need for strong external forces to activate more slip systems. The TD samples exhibit the highest yield strength (1015 MPa) and tensile strength (1105 MPa) among all directional samples. In the meantime, among the three directional samples, the RD samples exhibit moderate (centered) tensile properties. In particular, the yield strength (978 MPa) and ultimate tensile strength (1076 MPa) of the RD samples are closer to those of the TD specimens. Essentially, two main reasons can be proposed for the high strength of the TD specimens: (a) the basal <a> slip system is mostly in the hard orientation and is rarely activated, requiring strong external forces to produce deformation, and (b) many Pyramidal <c + a> and Pyramidal <a> slip systems are activated under low SF conditions, requiring the repression of large CRSSs. Therefore, the CRSS values during stretching are high, resulting in a high yield strength.

### 4.3. Influence of Texture and Slip Systems on the Mechanical Properties

**Figure 11:**
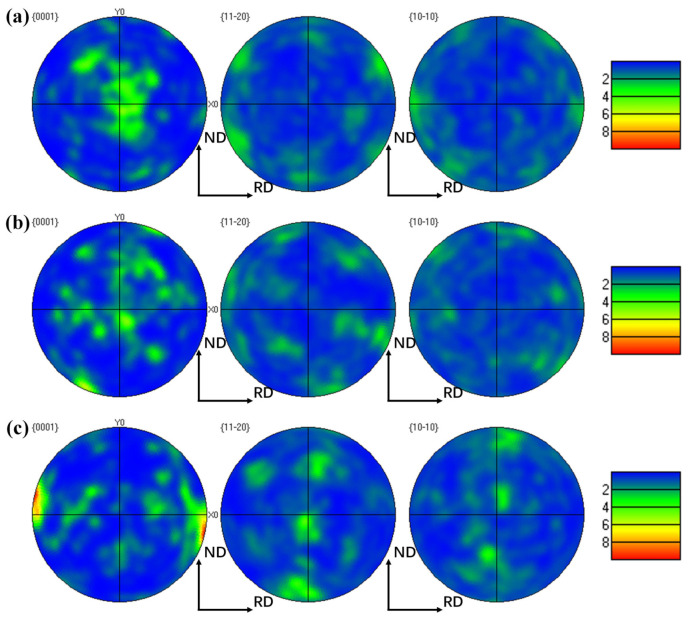
PF analysis of in situ tensile specimens: (**a**) ND; (**b**) RD; (**c**) TD.

**Figure 12:**
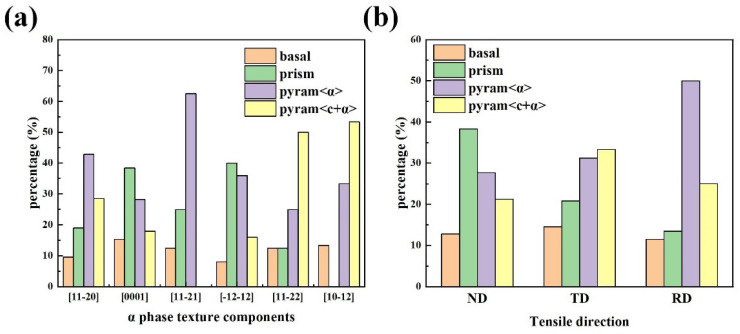
(**a**) Activated slip systems of the six α-phase texture components; (**b**) activated slip systems detected on the surfaces of the ND, RD, and TD samples.

The pole figure generated can indicate if texture formed during deformation; the texture of the α phase for the three directions is shown in Figure 11. A strong basal texture is dominant for the ND samples, and the textures for the TD and RD appear to have stronger intensity. Notably, the orientations of 12 α-phase variants precipitated from cubic-oriented β grains were obtained in a previous study [50]. Based on the above PF diagram conclusions and the main categories of the α-phase texture, six types of orientations with [11$\overset{-}{2}$0] as the primary ones were selected, and the proportions of activated slip systems were calculated (Figure 12a). Notably, the error between the ideal and actual orientations is strictly within 20°. Obviously, the Prismatic <a> slip system has an advantage in the [0001] texture, and few substrate <a> slips are observed in each orientation. Compared with those in the [$\overset{-}{1}$2$\overset{-}{1}$2] and [0001] orientations, the proportions of the Pyramid <α> and Pyramid <c + α> slips in the [11$\overset{-}{2}$0], [11$\overset{-}{2}$1], [11$\overset{-}{2}$2], and [10$\overset{-}{1}$2] orientations substantially increased.

The results of the SF and CRSS measurements can provide more information on the strength anisotropy. For the ND samples, the [0001] and [$\overset{-}{1}$2$\overset{-}{1}$2] texture are dominated by the Prismatic <a> slip. As the two slip orientations above are associated with low CRSS and high SF values, the ND samples have low yield strengths. The proportion of Pyramid <a> slips in the RD sample is high, attributed to the [$\overset{-}{1}$2$\overset{-}{1}$2] and [11$\overset{-}{2}$0] grain orientations. Additionally, due to the lower SF coefficient of the Pyramid <a> slip, under constant CRSS, the external force required to activate this slip system is greater than those for other slip systems, resulting in a slightly higher yield strength and tensile strength for the RD samples. Compared to the [0001] orientations of the ND and RD samples, the SF coefficients of the dominant slip system in the TD samples with the [11$\overset{-}{2}$2] and [10$\overset{-}{1}$2] orientations are relatively low. In addition, an increase in the slip ratio of the Pyramid < c + a> system implies a high CRSS value. Therefore, low SF and high CRSS values account for the comparatively high yield strength of the TD samples. The differences in activated slip systems with different textures lead to strength anisotropy, which is due to the differences in the SF and CRSS values.

As shown in Figure 9b and Table 7, for the RD and ND samples, the main activated slip modes of the α phase are Prismatic <a> and Pyramid <a>, respectively. For the TD samples, the main slip modes are Pyramid <a> and Pyramid <c + a>. Considering the CRSS, for the ND specimens, the yield stress caused by the Prismatic slip is the lowest. For the TD specimens, the activation of the Pyramid <c + a> slip requires comparatively strong external forces. The moderate yield strength of the RD specimen is attributed to its primary Pyramid <a> slip system. This indicates that the mechanical property anisotropy can be explained by the difference in slip activation.

## 5. Conclusions

This paper in situ investigated the effect of texture in TC11 forging on the deformation mechanism in the ND, RD, and TD through the EBSD technique. The slip behavior of constituent phases and the CRSS ratio of the α phase for Ti-6.5Al-3.5Mo-1.5Zr-0.3Si (TC11) alloy isothermally forged in the dual-phase region were investigated. The strength anisotropy mechanism is revealed by the SF and CRSS of dominated slip systems for different tensile samples. The following conclusions can be drawn.

(1) After annealing, TC11 forgings still exhibit considerable anisotropy of their tensile properties; in addition, the yield and ultimate tensile strengths of the samples decrease in the order of TD > RD > ND.

(2) There are differences in the activation slip systems of the RD, ND, and TD samples. The ND samples primarily comprise Prismatic slip systems, the RD samples primarily comprise Pyramidal <a> slip systems, and the TD samples comprise many Pyramidal <c + a> slip systems.

(3) The resistance coefficient (χ) is adopted to effectively evaluate the mechanical property anisotropy of the ND, TD, and RD specimens. The fundamental reason for the high strength of the TD samples is the hard orientations of the substrat, as well as the strong external forces required to activate the first-order and second-order Pyramid slips.

(4) In the ND and RD samples with the [0001] and [$\overset{-}{1}$2$\overset{-}{1}$2] texture components, mainly Prismatic <a> and basal <a> slips with high SF and low CRSS values are activated, resulting in relatively low yield strengths. However, for the TD samples comprising mainly [11$\overset{-}{2}$2] and [10$\overset{-}{1}$2] texture, high stress is required to activate the Pyramidal <a> and Pyramidal <c + a> slips with large CRSS values. Overall, the results confirm that the yield strength of the TD specimens is higher than those of the ND and RD specimens.

## 6. Limitations

The empirical results reported herein should be considered in the light of some limitations. The first is that the values of CRSS ratios differ from one study to another, which can be attributed to the microstructure studied and its morphology, grain sizes, phase proportions, or even its texture. The second limitation concerns the lack of previous research studies on the topic and the insufficient sample size for statistical measurement. Nonetheless, these results must be interpreted with caution, and a number of limitations should be borne in mind. In spite of their limitations, the experimental results are in line with the general law and are applicable to the research and industrial production of TC11 titanium alloy.

## Figures and Tables

**Table 1:** Identified slip systems and the Schmid factors (SFs) along the ND direction.

	Grain	Slip Plane and Direction	Activated System	SF
Stage I	1	($10\overset{-}{1}0$) [$1\overset{-}{2}10$]	Prismatic <a>	0.41
	2	(0001) [1$\overset{-}{2}10$]	Basal <a>	0.28
	3	($\overset{-}{1}\overset{-}{1}22)\;[11\overset{-}{2}3$]	Pyramidal slip <c + a>	0.33
	4	($1\overset{-}{1}00$) [11$\overset{-}{2}0$]	Prismatic <a>	0.35
	5	($1\overset{-}{1}00$) [11$\overset{-}{2}0$]	Prismatic <a>	0.40
	6	($10\overset{-}{1}0)\;[1\overset{-}{2}10$]	Prismatic <a>	0.38
	7	($\overset{-}{1}\overset{-}{1}22)\;[\overset{-}{1}\overset{-}{1}23$]	Pyramidal slip <c + a>	0.36
	8	($1\overset{-}{1}00$) [11$\overset{-}{2}0$]	Prismatic <a>	0.42
	9	($\overset{-}{2}112)\;[2\overset{-}{1}\overset{-}{1}3$]	Pyramidal slip <c + a>	0.32
	10	($10\overset{-}{1}0)\;[1\overset{-}{2}10$]	Prismatic <a>	0.41
Stage II	1	(0$\overset{-}{1}11$) [11$\overset{-}{2}0$]	Pyramidal slip <a>	0.35
	2	($\overset{-}{1}\overset{-}{1}22)\;[\overset{-}{1}\overset{-}{1}23$]	Pyramidal slip <c + a>	0.19
	3	($\overset{-}{2}112)\;[2\overset{-}{1}\overset{-}{1}3$]	Pyramidal slip <c + a>	0.26
	4	($\overset{-}{1}2\overset{-}{1}2)\;[1\overset{-}{2}13$]	Pyramidal slip <a>	0.36
	5	($1\overset{-}{1}00$) [11$\overset{-}{2}0$]	Prismatic <a>	0.37
	6	($10\overset{-}{1}1)\;[1\overset{-}{2}10$]	Pyramidal slip <a>	0.45
	7	($\overset{-}{1}\overset{-}{1}22)\;[11\overset{-}{2}3$]	Pyramidal slip <c + a>	0.37
	8	($\overset{-}{2}112)\;[2\overset{-}{1}\overset{-}{1}3$]	Pyramidal slip <c + a>	0.24
	9	($0\overset{-}{1}11$) [11$\overset{-}{2}0$]	Pyramidal slip <a>	0.33
	10	($10\overset{-}{1}0)\;[1\overset{-}{2}10$]	Prismatic <a>	0.49
	11	(0001) [1$\overset{-}{2}10$]	Basal <a>	0.45
	12	($1\overset{-}{1}00$) [11$\overset{-}{2}0$]	Prismatic <a>	0.32
	13	($1\overset{-}{1}00$) [11$\overset{-}{2}0$]	Prismatic <a>	0.28
	14	($10\overset{-}{1}0)\;[1\overset{-}{2}10$]	Prismatic <a>	0.47
	15	(0001) [11$\overset{-}{2}0$]	Basal <a>	0.41
	16	($10\overset{-}{1}0)\;[1\overset{-}{2}10$]	Prismatic <a>	0.06
	17	($10\overset{-}{1}0)\;[\overset{-}{2}110$]	Prismatic <a>	0.06
	18	($10\overset{-}{1}0)\;[1\overset{-}{2}10$]	Prismatic <a>	0.16
	19	($\overset{-}{1}101$) [11$\overset{-}{2}0$]	Pyramidal slip < a>	0.45
	20	($0\overset{-}{1}11$) [11$\overset{-}{2}0$]	Pyramidal slip <a>	0.44
	21	($01\overset{-}{1}0)\;[\overset{-}{2}110$]	Prismatic <a>	0.40
	22	($\overset{-}{2}112)\;[2\overset{-}{1}\overset{-}{1}3$]	Pyramidal < slip c + a>	0.27
	23	($1\overset{-}{1}01$) [11$\overset{-}{2}0$]	Pyramidal slip <a>	0.38
	24	($\overset{-}{1}2\overset{-}{1}2)\;[1\overset{-}{2}13$]	Pyramidal slip <a>	0.28
	25	($\overset{-}{2}112)\;[2\overset{-}{1}\overset{-}{1}3$]	Pyramidal slip <c + a>	0.37
	26	($\overset{-}{1}101$) [11$\overset{-}{2}0$]	Pyramidal slip <a>	0.44
	27	($10\overset{-}{1}0)\;[\overset{-}{2}110$]	Prismatic <a>	0.47
	28	($1\overset{-}{1}01$) [11$\overset{-}{2}0$]	Pyramidal slip <a>	0.41
	29	($\overset{-}{1}\overset{-}{1}22)\;[\overset{-}{1}\overset{-}{1}23$]	Pyramidal slip <c + a>	0.31
	30	($0\overset{-}{1}11$) [11$\overset{-}{2}0$]	Pyramidal slip <a>	0.47
	31	($01\overset{-}{1}0)\;[\overset{-}{2}110$]	Prismatic <a>	0.07
	32	($10\overset{-}{1}0)\;[\overset{-}{2}110$]	Prismatic <a>	0.02
	33	($\overset{-}{1}2\overset{-}{1}2)\;[1\overset{-}{2}13$]	Pyramidal slip <a>	0.35
	34	($\overset{-}{1}\overset{-}{1}22)\;[\overset{-}{1}\overset{-}{1}23$]	Pyramidal slip <c + a>	0.38
	35	($\overset{-}{1}2\overset{-}{1}2)\;[1\overset{-}{2}13$]	Pyramidal slip <c + a>	0.39
	36	($\overset{-}{1}101$) [11$\overset{-}{2}0$]	Pyramidal slip <a>	0.33

**Table 2:** Identified slip systems and the Schmid factors (SFs) along the RD direction.

	Grain	Slip Plane and Direction	Activated System	SF
Stage I	1	($\overset{-}{1}101$) [11$\overset{-}{2}0$]	Pyramidal slip <a>	0.32
	2	($01\overset{-}{1}1)\;[\overset{-}{2}110$]	Pyramidal slip <a>	0.46
	3	($10\overset{-}{1}0)\;[1\overset{-}{2}10$]	Prismatic <a>	0.31
	4	($01\overset{-}{1}1)\;[\overset{-}{2}110$]	Pyramidal slip <a>	0.25
	5	($01\overset{-}{1}1)\;[\overset{-}{2}110$]	Pyramidal slip <a>	0.46
	6	($01\overset{-}{1}1)\;[\overset{-}{2}110$]	Pyramidal slip <a>	0.24
	7	($10\overset{-}{1}1$) [1$\overset{-}{2}10$]	Pyramidal slip <a>	0.42
	8	($\overset{-}{1}\overset{-}{1}22)\;[\overset{-}{1}\overset{-}{1}23$]	Pyramidal slip <c + a>	0.27
	9	($1\overset{-}{1}00$) [11$\overset{-}{2}0$]	Prismatic <a>	0.29
	10	(0001) [1$1\overset{-}{2}0$]	Basal <a>	0.42
	11	($\overset{-}{1}2\overset{-}{1}2$) [1$\overset{-}{2}13$]	Pyramidal slip <c + a>	0.27
	12	($\overset{-}{1}2\overset{-}{1}2)\;[1\overset{-}{2}13$]	Pyramidal slip <a>	0.28
	13	($10\overset{-}{1}1$) [1$\overset{-}{2}10$]	Pyramidal slip <a>	0.40
	14	($\overset{-}{1}2\overset{-}{1}2$) [1$\overset{-}{2}13$]	Pyramidal slip <c + a>	0.50
	15	($\overset{-}{2}112)\;[2\overset{-}{1}\overset{-}{1}3$]	Pyramidal slip <c + a>	0.37
	16	($\overset{-}{1}\overset{-}{1}22)\;[11\overset{-}{2}3$]	Pyramidal slip <c + a>	0.37
Stage II	1	($\overset{-}{2}112)\;[2\overset{-}{1}\overset{-}{1}3$]	Pyramidal slip <c + a>	0.21
	2	($\overset{-}{2}112)\;[2\overset{-}{1}\overset{-}{1}3$]	Pyramidal slip <c + a>	0.42
	3	($10\overset{-}{1}0)\;[1\overset{-}{2}10$]	Prismatic <a>	0.41
	4	($0\overset{-}{1}11$) [11$\overset{-}{2}0$]	Pyramidal slip <a>	0.45
	5	($01\overset{-}{1}1)\;[\overset{-}{2}110$]	Pyramidal slip <a>	0.42
	6	($01\overset{-}{1}1)\;[\overset{-}{2}110$]	Pyramidal slip <a>	0.46
	7	(0001) [1$\overset{-}{2}10$]	Basal <a>	0.21
	8	($2\overset{-}{1}\overset{-}{1}2)\;[\overset{-}{2}113$]	Pyramidal slip <c + a>	0.50
	9	($01\overset{-}{1}1)\;[\overset{-}{2}110$]	Pyramidal slip <a>	0.44
	10	($\overset{-}{1}\overset{-}{1}22)\;[\overset{-}{1}\overset{-}{1}23$]	Pyramidal slip <c + a>	0.31
	11	($01\overset{-}{1}1)\;[\overset{-}{2}110$]	Pyramidal slip <a>	0.44
	12	($\overset{-}{1}\overset{-}{1}22)\;[11\overset{-}{2}3$]	Pyramidal slip <c + a>	0.01
	13	($01\overset{-}{1}1)\;[\overset{-}{2}110$]	Pyramidal slip <a>	0.29
	14	($10\overset{-}{1}1$) [1$\overset{-}{2}10$]	Pyramidal slip <a>	0.37
	15	($0\overset{-}{1}11$) [11$\overset{-}{2}0$]	Pyramidal slip <a>	0.38
	16	($\overset{-}{1}101$) [11$\overset{-}{2}0$]	Pyramidal slip <a>	0.45
	17	(0001) [1$\overset{-}{2}10$]	Basal <a>	0.02
	18	(0001) [1$1\overset{-}{2}0$]	Basal <a>	0.07
	19	($0\overset{-}{1}11$) [11$\overset{-}{2}0$]	Pyramidal slip <a>	0.33
	20	($1\overset{-}{1}00$) [11$\overset{-}{2}0$]	Prismatic <a>	0.50
	21	($10\overset{-}{1}1$) [1$\overset{-}{2}10$]	Pyramidal slip <a>	0.38
	22	($01\overset{-}{1}1)\;[\overset{-}{2}110$]	Pyramidal slip <a>	0.36
	23	($10\overset{-}{1}1$) [1$\overset{-}{2}10$]	Pyramidal slip <a>	0.35
	24	($1\overset{-}{1}00$) [11$\overset{-}{2}0$]	Prismatic <a>	0.34
	25	($1\overset{-}{1}00$) [11$\overset{-}{2}0$]	Prismatic <a>	0.26
	26	($0\overset{-}{1}11$) [11$\overset{-}{2}0$]	Pyramidal slip <a>	0.25
	27	(0001) [1$1\overset{-}{2}0$]	Basal <a>	0.35
	28	($0\overset{-}{1}11$) [11$\overset{-}{2}0$]	Pyramidal slip <a>	0.25
	29	($\overset{-}{1}\overset{-}{1}22)\;[11\overset{-}{2}3$]	Pyramidal slip <c + a>	0.44
	30	($1\overset{-}{1}01$) [11$\overset{-}{2}0$]	Pyramidal <a> slip	0.30
	31	($10\overset{-}{1}0)\;[1\overset{-}{2}10$]	Prismatic <a>	0.29
	32	($\overset{-}{1}\overset{-}{1}22)\;[\overset{-}{1}\overset{-}{1}23$]	Pyramidal slip <c + a>	0.32
	33	(0001) [1$1\overset{-}{2}0$]	Basal <a>	0.49
	34	($01\overset{-}{1}1)\;[\overset{-}{2}110$]	Pyramidal slip <a>	0.16
	35	($10\overset{-}{1}1$) [1$\overset{-}{2}10$]	Pyramidal slip <a>	0.35
	36	($\overset{-}{1}\overset{-}{1}22)\;[\overset{-}{1}\overset{-}{1}23$]	Pyramidal slip <c + a>	0.39

**Table 3:** Identified slip systems and the Schmid factors (SFs) along the TD direction.

	Grain	Slip Plane and Direction	Activated System	SF
Stage I	1	($2\overset{-}{1}\overset{-}{1}2)\;[\overset{-}{2}113$]	Pyramidal slip <c + a>	0.47
	2	($1\overset{-}{1}00$) [11$\overset{-}{2}0$]	Prismatic <a>	0.42
	3	(0001) [$\overset{-}{2}110$]	Basal <a>	0.31
	4	($1\overset{-}{1}00$) [11$\overset{-}{2}0$]	Prismatic <a>	0.41
	5	($1\overset{-}{1}00$) [11$\overset{-}{2}0$]	Prismatic <a>	0.50
	6	($\overset{-}{2}112)\;[2\overset{-}{1}\overset{-}{1}3$]	Pyramidal slip <c + a>	0.25
	7	($\overset{-}{2}112)\;[2\overset{-}{1}\overset{-}{1}3$]	Pyramidal slip <c + a>	0.39
	8	($\overset{-}{2}112)\;[2\overset{-}{1}\overset{-}{1}3$]	Pyramidal slip <c + a>	0.44
	9	($\overset{-}{1}\overset{-}{1}22$) [11$\bar{2}3$]	Pyramidal slip <c + a>	0.50
	10	($\overset{-}{1}101$) [11$\overset{-}{2}0$]	Pyramidal slip <a>	0.40
	11	($10\overset{-}{1}1$) [1$\overset{-}{2}10$]	Pyramidal slip <a>	0.38
	12	($10\overset{-}{1}1$) [1$\overset{-}{2}10$]	Pyramidal slip <a>	0.34
Stage II	1	(0$\overset{-}{1}11$) [11$\overset{-}{2}0$]	Pyramidal slip <a>	0.42
	2	(0001) [11$\overset{-}{2}0$]	Basal <a>	0.30
	3	(0001) [11$\overset{-}{2}0$]	Basal <a>	0.45
	4	(0001) [$\overset{-}{2}110$]	Basal <a>	0.21
	5	($01\overset{-}{1}1)\;[\overset{-}{2}110$]	Pyramidal slip <a>	0.42
	6	($1\overset{-}{1}00$) [11$\overset{-}{2}0$]	Prismatic <a>	0.44
	7	($\overset{-}{1}\overset{-}{1}22$) [11$\bar{2}3$]	Pyramidal slip <c + a>	0.49
	8	($1\overset{-}{1}01$) [11$\overset{-}{2}0$]	Pyramidal slip <a>	0.31
	9	($1\overset{-}{1}00$) [11$\overset{-}{2}0$]	Prismatic <a>	0.35
	10	($\overset{-}{1}\overset{-}{1}22)\;[\overset{-}{1}\overset{-}{1}23$]	Pyramidal slip <c + a>	0.31
	11	($1\overset{-}{1}01$) [11$\overset{-}{2}0$]	Pyramidal slip <a>	0.45
	12	($2\overset{-}{1}\overset{-}{1}2)\;[\overset{-}{2}113$]	Pyramidal slip <c + a>	0.46
	13	($\overset{-}{1}2\overset{-}{1}2$) [1$\overset{-}{2}13$]	Pyramidal slip <c + a>	0.41
	14	($0\overset{-}{1}11$) [11$\overset{-}{2}0$]	Pyramidal slip <a>	0.48
	15	(0001) [$\overset{-}{2}110$]	Basal <a>	0.41
	16	(0$1\overset{-}{1}0)\;[\overset{-}{2}110$]	Prismatic <a>	0.42
	17	($2\overset{-}{1}\overset{-}{1}2)\;[\overset{-}{2}113$]	Pyramidal slip <c + a>	0.33
	18	($\overset{-}{1}\overset{-}{1}22)\;[\overset{-}{1}\overset{-}{1}23$]	Pyramidal slip <c + a>	0.23
	19	($01\overset{-}{1}1)\;[\overset{-}{2}110$]	Pyramidal slip <a>	0.40
	20	(0001) [$1\overset{-}{2}10$]	Basal <a>	0.41
	21	($\overset{-}{2}112)\;[2\overset{-}{1}\overset{-}{1}3$]	Pyramidal slip <c + a>	0.38
	22	(0$1\overset{-}{1}0)\;[\overset{-}{2}110$]	Prismatic <a>	0.28
	23	($\overset{-}{1}2\overset{-}{1}2)\;[1\overset{-}{2}13$]	Pyramidal slip <a>	0.34
	24	($10\overset{-}{1}0)\;[\overset{-}{2}110$]	Prismatic <a>	0.46
	25	($10\overset{-}{1}0)\;[\overset{-}{2}110$]	Prismatic <a>	0.45
	26	($1\overset{-}{1}00$) [11$\overset{-}{2}0$]	Prismatic <a>	0.41
	27	(0001) [$\overset{-}{2}110$]	Basal <a>	0.28
	28	($1\overset{-}{1}01$) [11$\overset{-}{2}0$]	Pyramidal slip <a>	0.45
	29	($\overset{-}{2}112)\;[2\overset{-}{1}\overset{-}{1}3$]	Pyramidal slip <c + a>	0.21
	30	($\overset{-}{1}\overset{-}{1}22)\;[\overset{-}{1}\overset{-}{1}23$]	Pyramidal slip <c + a>	0.49
	31	($\overset{-}{1}\overset{-}{1}22$) [11$\bar{2}3$]	Pyramidal slip <c + a>	0.39
	32	($01\overset{-}{1}1)\;[\overset{-}{2}110$]	Pyramidal slip <a>	0.35
	33	($\overset{-}{1}2\overset{-}{1}2)\;[1\overset{-}{2}13$]	Pyramidal slip <a>	0.33
	34	($\overset{-}{2}112)\;[2\overset{-}{1}\overset{-}{1}3$]	Pyramidal slip <c + a>	0.47
	35	($01\overset{-}{1}1)\;[\overset{-}{2}110$]	Pyramidal slip <a>	0.35
	36	($10\overset{-}{1}1$) [1$\overset{-}{2}10$]	Pyramidal slip <a>	0.33

**Table 4:** Favorable slip systems of TC11 titanium forging in yielding stage and hardening stage.

Favorable Slip Systems	Yielding Stage	Hardening Stage
ND	Basal <a> slips, prismatic <a> slips, Pyramidal < c + a> slips	Pyramidal < a> slips
RD	prismatic <a> slips, Pyramidal < a> slips, Pyramidal < c + a> slips	Basal < a> slips
TD	Basal < a> slips, prismatic <a> slips, Pyramidal < a> slips Pyramidal < c + a> slips	No newly activated slip system

**Table 5:** CRSS ratio results and comparison with the literature.

Alloy Used	Basal <a>	Pyramidal <c + a>	Reference
Ti-6242S	0.82–1.2	4.08–5.78	[36]
Ti-6242S	1.06	1.80	[37]
Ti-6Al-4V	1–1.5	3.0–3.5	[38]
Ti-6Al-4V	1.33–1.41	2.72–3.41	[39]
Ti-6Al-4V	0.93–1.3	1.1–1.6	[40]
Ti-6Al-4V	1.43	4.23	[41]
Ti-6Al-4V	0.93	1.80	[42]
Ti-6Al-4V	1.13	1.61	[43]
Ti-6Al-4V	1.14	1.59	[44]
Ti-6Al	1.01	2.64	[45]
Ti-6Al	0.95	3.85	[46]

**Table 6:** The Resistance Coefficient calculated along the ND, TD and RD direction.

Direction	$R_{\mu }^{basal}$	$R_{\mu }^{prism}$	$R_{\mu }^{pyram-1st}$	$R_{\mu }^{pyram-2st}$	$R_{f}^{basal}$	$R_{f}^{prism}$	$R_{f}^{pyram-1st}$	$R_{f}^{pyram-2st}$	χ
ND°	0.24	0.45	0.22	0.38	0.17	0.42	0.25	0.16	0.58
TD°	0.32	0.17	0.41	0.28	0.15	0.16	0.28	0.41	0.76
RD°	0.48	0.31	0.29	0.33	0.21	0.15	0.39	0.25	0.73

**Table 7:** SFs of the six α-phase texture components.

Slip System	$[11\overset{-}{2}0$]	[0001]	$[11\overset{-}{2}1$]	$[\overset{-}{1}2\overset{-}{1}2$]	$[11\overset{-}{2}2$]	$[10\overset{-}{1}2$]
Bas	0.35	0.29	0.35	0.31	0.31	0.14
Pri	0.31	0.41	0.45	0.43	0.28	0
Pyr I	0.38	0.37	0.47	0.37	0.34	0.36
Pyr II	0.34	0.40	0	0.32	0.41	0.39

## Data Availability

The original contributions presented in this study are included in the article. Further inquiries can be directed to the corresponding author.
